# Antibiotic prophylaxis in the prevention of urinary tract infection in patients with sterile urine before extracorporeal shock wave lithotripsy

**DOI:** 10.22088/cjim.9.3.296

**Published:** 2018

**Authors:** Hamdi Shafi, Masomeh Ilkhani, Zeinab Darabi Ahangar, Masomeh Bayani

**Affiliations:** 1 Infertility and Reproductive Health Research Center, Health Research Institute, Babol University of Medical Sciences, Babol, Iran; 2 Student Research Committee, Babol University of Medical Sciences, Babol, Iran; 3 Infectious Diseases and Tropical Medicine Research Center, Health Research Institute, Babol University of Medical Sciences, Babol, Iran

**Keywords:** ESWL, Prophylactic antibiotic, Urine culture

## Abstract

**Background:**

One of the lithotripsy complications is urinary tract infection (UTI) and sepsis after extracorporeal shock wave lithotripsy (ESWL). The aim was to study the prophylactic effect of antibiotics on UTI after ESWL.

**Methods:**

This randomized double-blind clinical trial was carried out on 600 patients admitted to Babol Clinic hospital in 2014-2015. Patients were randomly divided into treatment group (receiving 200 mg ofloxacin and control group (receiving placebo. The effect of prophylactic antibiotics on the incidence of bacteriuria after ESWL and the impact of variables such as gender, age, urolithiasis size and location and underlying diseases in the incidence of UTI after ESWL were evaluated.

**Results:**

Totally, 67 of the population had positive urine cultures. Twenty-nine (10.13%) of them were in the treatment group (n=286) and 38 (13.01%) of them were in the control group (n=292). All 67 patients had asymptomatic bacteriuria. Escherichia coli and proteus were the grown microorganisms in most samples. The mean age of sample population was 44.8±23, and 67.16% of patients with positive urine culture were older than 45 years.

**Conclusions:**

The results indicated that prophylactic antibiotics prior to ESWL in patients with *urinary calculi* and negative urine culture had no significant decrease in urinary tract infection after lithotripsy. It is better that the use of prophylactic antibiotics is limited to patients with risk factors.

Extracorporeal shock wave lithotripsy (ESWL) is a non-invasive method for the treatment of urolithiasis and choledocholithiasis using acoustic waves (1, 2). One of the side effects of this method is urinary tract infection (UTI) after lithotripsy and sepsis in severe cases (3, 4). There are several studies in different countries regarding the incidence of UTI after lithotripsy and the use of antibiotic prophylaxis, which have no consensus on the use of antibiotic prophylaxis. *American Urological Association* (AUA) states that the use of prophylactic antibiotics is necessary based on the patient's condition or the type of surgery resulting in UTI (5), while *European Association of Urology*** (**EAU) believes that the use of prophylactic antibiotics is necessary only for proven prostate biopsy and *transurethral resection* of the prostate (TURP) (6). Therefore, the aim of the current study was to evaluate the efficacy of prophylactic antibiotics in patients undergoing lithotripsy and study the risk factors predisposing to UTI after lithotripsy. 

## Methods

Methods

This randomized double-blind clinical trial was approved by the Ethics Committee of Babol University of Medical Sciences *(*IRCT201506242897N1). 

Sampling was done during nine months, from December 2014 to August 2015. The sample consisted of 600 patients who referred to Babol Clinic hospital after the diagnosis of urolithiasis using kidney, ureter and bladder (KUB) to break up stones. Inclusion criteria were; patients with the age of 18 years or older, negative urine culture before ESWL (<10^5^ bacterial colonies per ml), the absence of a foley catheter or nephrostomy tube. Patients were randomly divided into two groups (treatment and control). The treatment group received ofloxacin tablets (200 mg/12 h/3 days) after operation and the control group took placebo made of flour. Two weeks after ESWL, *ultrasound* was performed to examine the remains of the detectable stones for all patients. Information related to the symptomatic UTI was recorded. 

Twenty-two patients who did not refer for urine culture two weeks after ESWL or who had endourological manipulations during and after ESWL were excluded from the present study. Finally, data were analyzed using SPSS16 through statistical tests such as *multivariate chi****-****square***,** logistic regression and t-test. 

## Results

According to the results of urine culture performed two weeks after the operation, 67 of the population had positive urine culture. Twenty-nine (10.13%) and 38 (13.01%) of these 67 patients were in treatment and control groups (P=0.082), respectively. All 67 patients had asymptomatic bacteriuria without urosepsis. Escherichia coli and proteus were the grown microorganisms in most samples. The mean age of sample population was 44.8±23 years and 67.16% of patients with positive urine culture were older than 45 years. In addition, 19.4% of patients with positive urine culture had underlying disease of diabetes and 20.8% of them had a history of UTI (p=0.03, 0.015, respectively). Moreover, 29.8% patients with positive urine culture had a history of the transurethral lithotomy (TUL) and surgeries of the urinary system within 5 years. Nevertheless, there was no significant relationship between hypertension, a history of TUL and TURP with bacteriuria. Male to female ratio was 4 to 5 in patients with positive urine culture. Averagely, the location of urolithiasis in 46.2% of patients with positive urine culture was in kidney (P=0.11). Furthermore, 58.2% of patients with positive urine culture had urinary stones with a diameter of 10-19 mm and 19.4% of patients with urinary stones greater than 20 mm in diameter. There was a significant relationship between bacteriuria after lithotripsy and a diameter of urolithiasis (p=0.013). Moreover, the risk factors were diabetes (p=0.004), stone size (p=0.03) and age (66-85, p=0.011).

## Discussion

In the current study, the incidence of bacteriuria was 10.13% and 13.01% in the treatment and control groups, respectively. The incidence of bacteriuria after ESWL was generally low in patients and the use of antibiotic prophylaxis had no significant difference to reduce the incidence of bacteriuria after ESWL. In a study of Moreno et al., culture was positive in 8.5% of patients 7 days after ESWL, so 2.1% of these patients were symptomatic and the rest were asymptomatic. They did not use any antibiotics in their study so the statistical results of both studies could not be compared in terms of the effect of prophylactic antibiotics (7). Infectious complications were found in 1/3% of patients in a study of Honey et al. who assessed the need of antibiotic prophylaxis before ESWL (8). Therefore, it is clear that antibiotic prophylaxis before ESWL is not necessary in patients without risk factors and with negative urine culture. The effect of prophylaxis antibiotic is debatable in male elderly patients. Bacteriuria after ESWL was higher in men over 45 years in the present study, which differs from the result of Alexander Cameron et al. in terms of gender and resembles their result in terms of age. Bacteriuria was higher in women and older patients in their study (9).

One reason for the high incidence of bacteriuria in elderly men in our study was the high number of men in both groups. Moreover, the high probability of benign prostatic hypertrophy and its role in urinary stasis in creating fertile ground for bacteriuria in older men can be reasonable causes in the present study. 

To avoid these complications, some strategies such as removing the previous underlying disease, early treatment of UTI, using prophylaxis antibiotic and reducing the number and energy of shock waves had been proposed (10). Their results of the determination of underlying diseases including previous UTI as a risk factor for creating UTI after ESWL and the need of antibiotic prophylaxis in patients with risk factors are similar to those of the current study. In the current study, in terms of the location of *urinary calculi* and its role in the incidence of bacteriuria after ESWL, it was concluded that in the majority of patients in both groups, the location of stone was in kidney and upper ureter, but the role of stone location has not been proven in the incidence of bacteriuria yet. In terms of stone size and its role in the incidence of bacteriuria after ESWL in the present study, it was suggested that the diameter of urolithiasis was between 11-19 mm in the majority of patients (58.2%) and the highest incidence of bacteriuria after ESWL was observed in patients with stones over 11 mm. Moreno et al. on evaluating the risk factor of stone size showed that the rate of bacteriuria after ESWL was higher in patients with greater diameter of stone (7). The higher incidence of bacteriuria in larger stones can be attributed to the high number of crushed stones after ESWL and more likely of creating minor damages on the endothelial surfaces by crushed stones and release of higher levels of bacteria of stones. In several studies, it was suggested that the use of antibiotic prophylaxis had little effect on preventing from the incidence of bacteriuria after lithotripsy; therefore, an appropriate use of antibiotics can not only reduce the drug complications but also prevent the resistance of organisms against these drugs (9, 11-13).

In conclusion, the results of the current study suggested that the rate of infectious complications after ESWL had no significant reduction with the use of prophylactic antibiotics and generally, infectious complications were higher in older men and those with a history of diabetes, UTI and stones larger than 10 mm in upper ureteral. The use of prophylactic antibiotics is recommended in patients with one or more mentioned risk factors.
